# Risk of Radiation-Associated Contralateral Breast Cancer in Germline Mutation Carriers: A Meta-Analysis and Systematic Review

**DOI:** 10.3390/cancers18071106

**Published:** 2026-03-29

**Authors:** Christina Hari Nawangsih Prihharsanti, Yan Wisnu Prajoko, Danendra Rakha Putra Respati, Kevin Christian Tjandra, Fitri Mutmainnah, Maritza Bintang Rismadha, Annisa Salsabilla Dwi Nugrahani, Davendra Putra Aryasatya, Andrea Valerie Manik, Fahrul Nurkolis, Edward Kurnia Setiawan Limijadi

**Affiliations:** 1 Division of Radiation Oncology, Department of Radiology, Faculty of Medicine, Universitas Diponegoro, Semarang 50275, Indonesia; cnawangsih@lecturer.undip.ac.id; 2 Department of Oncology Surgery, Faculty of Medicine, Universitas Diponegoro, Semarang 50275, Indonesia; yanprajoko7519@gmail.com; 3 Faculty of Medicine, Universitas Diponegoro, Semarang 50275, Indonesia; rakhapres@gmail.com (D.R.P.R.); kevinchristian2841@gmail.com (K.C.T.); maritzabintangrismadha@gmail.com (M.B.R.); vendra.dap@gmail.com (D.P.A.); 4 Faculty of Medicine, Universitas Andalas, Padang 25163, Indonesia; fitrimutmainnah04@gmail.com; 5 Medical Science in Clinical Investigation, Harvard Medical School, Boston, MA 02115, USA; annisa_nugrahani@hms.harvard.edu; 6 Oncology Research, University of Manchester, Manchester M13 9PL, UK; manik.andrea@gmail.com; 7 Program of Basic Medical Science, Faculty of Medicine, Universitas Airlangga, Surabaya 60131, Indonesia; fahrul.nurkolis.mail@gmail.com; 8 Medical Research Center of Indonesia, Surabaya 60281, Indonesia; 9 Institute for Research and Community Service, State Islamic University of Sunan Kalijaga (UIN Sunan Kalijaga), Yogyakarta 55281, Indonesia; 10 Department of Clinical Pathology, Faculty of Medicine, Universitas Diponegoro, Semarang 50275, Indonesia

**Keywords:** contralateral breast cancer, radiotherapy, germline mutations, BRCA1/2, CHEK2, ATM

## Abstract

**Background/Objectives**: Contralateral breast cancer (CBC) is a significant concern among breast cancer survivors, particularly in those with moderator-high penetrance germline mutations such as BRCA1, BRCA2, CHEK2, and ATM. While radiotherapy (RT) is a crucial component of breast cancer (BC) treatment, its potential role in increasing CBC risk remains unclear. This systematic review and meta-analysis aim to evaluate the incidence of radiation-induced CBC in germline mutation carriers. **Methods**: Following PRISMA guidelines, we conducted a comprehensive search in six electronic databases (PubMed, Scopus, Cochrane Library, ProQuest, EBSCO, and Epistemonikos) for studies published fifteen years prior, up to August 2025. We included cohort and case–control studies assessing the association between RT and CBC incidence in germline mutation carriers. A meta-analysis was performed using a random-effects model to estimate cumulative risk (CR) and rate ratios (RR). **Results**: Seven studies were included. The 5-year cumulative risk (CR) of contralateral breast cancer (CBC) was 0.55 for BRCA1/2, 0.89 for ATM, and 0.80 for CHEK2 carriers. At 10 years, overall CR increased to 0.65, with ATM and CHEK2 remaining high. Rate ratio (RR) analysis showed a significant risk for ATM (2.98), while overall RR indicated more than a two-fold increased CBC risk with radiotherapy (RR = 2.70 common-effect; 2.53 random-effects). **Conclusions**: Radiotherapy significantly increases contralateral breast cancer risk, particularly in ATM and CHEK2 carriers, emphasizing the importance of personalized genetic risk stratification in treatment decisions.

## Simple Summary

Contralateral breast cancer is a new cancer that develops in the opposite breast after a woman has already been treated for breast cancer, and it is a particular concern in women who carry inherited gene changes such as BRCA1, BRCA2, CHEK2, or ATM. Radiotherapy is an important part of breast cancer treatment, but there is uncertainty about whether it increases the chance of a new cancer in the other breast among women with these genetic risks. In this study, we combined and re-analyzed data from several previous clinical studies to compare how often contralateral breast cancer occurs in mutation carriers who did and did not receive radiotherapy. Our results suggest that radiotherapy is associated with a higher risk of contralateral breast cancer in carriers of certain gene mutations, especially BRCA1/2 and CHEK2. These findings may help support more personalized treatment planning for women with hereditary breast cancer.

## 1. Introduction

Although contralateral breast cancer (CBC) occurs less frequently and poses a lower risk than the recurrence of initial tumor, this issue remains a significant challenge [1]. As a common second malignancy found in women with a history of primary BC, approximately 2 to 11% of women diagnosed with BC will develop CBC at some point in their lifetime [2]. There is a 1.5–5.5-fold risk of primary BC patients developing CBC compared to the general population [3]. Germline mutation status, race/ethnicity, age at diagnosis, and menopausal status of BC patients highly affect the risk of developing CBC [4].

One of the most common treatments for BC is radiotherapy (RT). It plays a crucial role in the comprehensive treatment, contributing to the management of early-stage, locally advanced, and metastatic cases [5]. Currently, early-stage primary breast cancer (BC) is commonly managed with breast-conserving surgery followed by adjuvant radiotherapy, as robust evidence—including the Early Breast Cancer Trialists’ Collaborative Group (EBCTCG) meta-analyses—has demonstrated that radiotherapy significantly reduces local recurrence and contributes to improved long-term breast cancer-specific outcomes [6]. Although most studies do not find a statistically significant increase in CBC risk among patients receiving breast RT compared to those treated with mastectomy alone, it is plausible that scattered or incidental radiation exposure to the contralateral breast, reaching doses of several grays, contributes to the overall risk of developing a second malignancy. While the overall risk appears minimal for most patients, a subset with genetic or environmental susceptibilities may be more vulnerable to developing radiation-associated CBC [6,7].

The genetic foundation of BC is associated with various high- and moderate-penetrance mutations in susceptibility genes. Among these, BRCA1, BRCA2, TP53, and ATM are the most extensively studied tumor suppressor genes, significantly influencing primary BC treatment strategies [8,9]. It is widely recognized that BC patients with BRCA mutations have a significantly increased risk of developing CBC. The risk is significantly higher in patients with BRCA1 mutations compared to those with BRCA2 mutations (*p* = 0.04) [10].

Deleterious germline variants play a significant role in breast cancer progression and the development of contralateral breast cancer (CBC), with evidence showing that CBC occurs approximately three times more frequently in BRCA mutation carriers (15.3%) compared to women with unilateral breast cancer (5.2%) [11]. At the molecular level, exposure to ionizing radiation activates key DNA damage response pathways, particularly through the ATM gene, which regulates cell cycle arrest, DNA repair, and apoptosis. Notably, women harboring rare, potentially pathogenic ATM missense variants have been shown to have a significantly increased risk of CBC following radiation exposure compared with both unexposed carriers and individuals with wild-type genotypes [12].

In parallel with advances in genetic risk profiling, recent developments in breast cancer detection have increasingly incorporated computational and imaging-based technologies. A recent meta-analysis by Leung et al. (2024) demonstrated that computer-aided detection using hyperspectral imaging can enhance diagnostic performance in breast cancer by improving lesion characterization and detection accuracy [12]. These findings highlight the growing role of artificial intelligence-assisted imaging in refining early detection and risk stratification, which may ultimately support more individualized clinical decision-making, particularly in patients with elevated genetic susceptibility.

The risk of CBC in carriers of other germline pathogenic variants (PVs), particularly CHEK2 and PALB2, remains unclear [4]. The CHEK2 gene produces a protein that plays a key role in regulating the cell cycle and repairing DNA damage. A specific truncating mutation, 1100delC, has been associated with a two-fold risk of developing BC, as identified in the CHEK2 Breast Cancer Case–Control Consortium pooling analysis [13]. Individuals carrying the CHEK2.1100delC mutation are believed to be more sensitive to treatments like RT and chemotherapy, which induce DNA double-strand breaks. This is because CHEK2 plays a crucial role in regulating cell-cycle checkpoint pathways that respond to DNA damage [14]. As for patients with PALB2 mutation, it is found that the adjusted hazard ratio (HR) for developing CBC was a 2.67-fold risk compared to the general population [15].

Although germline mutations in BRCA1/2, CHEK2, and ATM are well-recognized to increase the baseline risk of contralateral breast cancer (CBC), the extent to which radiotherapy (RT) further modifies this risk remains an important and unresolved clinical question. This uncertainty reflects a broader therapeutic challenge in hereditary breast cancer, where clinicians must balance the well-established benefits of RT in improving local control and survival against the potential long-term risk of radiation-induced secondary malignancies. In daily practice, this often leads to complex, individualized decision-making that incorporates genetic risk, tumor characteristics, patient preferences, and anticipated long-term outcomes.

However, the current body of evidence remains heterogeneous and at times conflicting, with limited mutation-specific analyses and variability in study design, radiation exposure assessment, and follow-up duration. As a result, the true magnitude of RT-associated CBC risk in genetically predisposed populations has not been clearly defined. In this context, our systematic review and meta-analysis aim to provide a more comprehensive synthesis of the available evidence, with a particular focus on germline mutation carriers. By integrating data across studies, this work seeks to clarify the potential association between RT and CBC risk and to better inform risk-adapted, individualized treatment strategies in this high-risk population.

## 2. Materials and Methods

### 2.1. Study Eligibility Criteria

This meta-analysis was conducted following the Preferred Reporting Items for Systematic Reviews and Meta-Analyses (PRISMA) guidelines [16]. The study protocol was registered in PROSPERO by 4th August 2025, with the registration number CRD420251116093. The inclusion and exclusion criteria were established a priori to the conducted search to ensure homogeneity among the included studies.

The inclusion criteria consisted of studies that (1) were published in English and provided accessible data on the topic; (2) employed a cohort or case–control study design; (3) included adult patients aged ≥18 years diagnosed with unilateral breast cancer (BC) carrying germline mutations (e.g., BRCA1/2, CHEK2, or ATM); and (4) evaluated the association between radiotherapy (RT) and the incidence of contralateral breast cancer (CBC), reporting quantitative effect estimates such as odds ratios (ORs), relative risks (RRs), or hazard ratios (HRs).

Importantly, studies were included only if patients with prior contralateral prophylactic mastectomy (CPM) were either excluded or clearly accounted for in the analysis, given that CPM eliminates the risk of CBC and may confound outcome assessment.

The exclusion criteria encompassed the following: (1) non-human studies; (2) case reports, reviews, editorials, conference abstracts, and non-peer-reviewed articles; (3) studies lacking a comparison group (RT vs. no RT); (4) studies that did not report outcomes related to CBC incidence; and (5) studies in which the status of contralateral prophylactic mastectomy could not be determined or was not appropriately addressed.

The eligibility assessment was performed independently by multiple reviewers (KCT, DRPR, FM, LAKN, and NRAW). Any discrepancies were resolved through discussion or consultation with senior reviewers (CHNP, YWP, EKSL).

### 2.2. Literature Search Strategy

A comprehensive literature search was conducted across six electronic databases, including PubMed, Scopus, the Cochrane Library, ProQuest, EBSCO, and Epistemonikos. The search encompassed studies published between 16 August 2015 and 16 August 2025. To ensure a systematic and reproducible approach, predefined search strategies were applied using combinations of relevant keywords and Boolean operators, including the following: (“BRCA” OR “CHEK2” OR “ATM” OR “germline mutation”) AND (“radiotherapy” OR “radiation therapy”) AND (“breast cancer” OR “contralateral breast cancer” OR “second primary”) AND (“clinical outcomes” OR “pathological outcomes” OR “tumor characteristics” OR “tumor progression” OR “therapeutic response”).

The study selection process was conducted in accordance with the Preferred Reporting Items for Systematic Reviews and Meta-Analyses (PRISMA) guidelines. After removing duplicate records, titles and abstracts were independently screened against predefined inclusion and exclusion criteria. Full-text articles were then retrieved and assessed for eligibility. This process was performed independently by four reviewers (DRPR, FM, LAKN, and MBR) to minimize selection bias. Any discrepancies were resolved through discussion and consensus among all authors.

In addition, the methodological quality and risk of bias of the included studies were evaluated using appropriate, study design-specific tools (ROBINS-E), allowing for a more structured appraisal of the reliability and validity of the pooled evidence. The overall study selection process is illustrated in the PRISMA flow diagram, and detailed results of the risk-of-bias assessment are presented in the corresponding section.

### 2.3. Data Extraction

Two independent authors (DRPR, KCT) extracted the following information from each study for analysis: study ID, year of publication, country, study design, sample size, germline genes, mean age of subjects, number of subjects in the RT and control groups, Estrogen Receptor (ER) and Human Epidermal Growth Factor Receptor 2 (HER2) status. All differences were addressed by engaging in discussions with the three validators (CHNP, YWP, EKSL). Extracted data were systematically recorded using Microsoft Excel 2021 and presented in Supplementary File S1. Baseline characteristics and outcome measures extracted in this study included the incidence and CR of CBC in patients with RT and No-RT exposure, as well as subgroup analyses for different germline mutations (BRCA1/2, CHEK2, ATM). Relevant data were extracted from included studies, which reported CR at 5 years and 10 years in both RT and No-RT groups as percentages along with their respective 95% confidence intervals (CIs). Additionally, RR values and their CIs were extracted where available.

### 2.4. Statistical Analysis

A meta-analysis was conducted to evaluate CR differences and rate ratios (RR) associated with RT for different genetic subgroups (BRCA1/2, CHEK2, ATM). Statistical computations were performed using RStudio version R 4.5.1 with the “meta” and “metafor” packages. For CR analysis, CR values were transformed into the logit scale using the logit function, and standard errors (SE) were calculated using the following: SE = (logit(upper/100) − logit(lower/100))/(2 × 1.96).

The logit risk difference between RT and non-RT groups was computed along with the combined SE for meta-analysis. A random-effects model using the DerSimonian–Laird (DL) estimator was applied. Heterogeneity was assessed using the I-squared (I^2^) statistic and Cochran’s Q test. Subgroup analyses were performed based on genetic mutation, and results were visualized in forest plots. Statistical significance was evaluated using Z-tests with corresponding *p*-values. Publication bias was examined using funnel plots and Egger’s regression test. For RR meta-analysis, RR values were log-transformed, and SE was calculated as the following: SE = (log(CI_high) − log(CI_low))/(2 × 1.96).

A subgroup analysis based on genetic mutations was performed, and results were displayed in a forest plot. The overall effect estimate and subgroup differences were tested using Z-tests, and heterogeneity was assessed with I^2^. A funnel plot was generated to evaluate potential publication bias. All statistical analyses were conducted in R-Studio with significance set at *p* < 0.05. Results were interpreted with caution, considering limitations of observational studies and study variability.

### 2.5. Quality Assessment

The Risk of Bias in Non-randomized Studies-of Exposures (ROBINS-E) tool was used to evaluate the risk of bias in the non-randomized studies that were part of this meta-analysis [17]. Seven domains of bias were used to evaluate each study: confounding, participant selection, intervention classification, deviations from intended interventions, missing data, outcome measurement, and selection of reported results. The results of the quality assessment were recorded in the “bias” column of a Microsoft Excel 2021 workbook. To provide a visual summary of the risk of bias, the recorded data were uploaded to the ROBVIS website https://mcguinlu.shinyapps.io/robvis/ (accessed on 2 September 2025), and the output was presented as a traffic light plot [18]. This systematic appraisal method enabled a transparent and detailed depiction of bias across the included studies, thereby strengthening the validity of the meta-analysis findings. The risk of bias’ full report is available as Supplementary File S2.

### 2.6. Certainty Assessment

To assess the certainty of evidence for each outcome, we used the Grading of Recommendations, Assessment, Development and Evaluations (GRADE) approach. This method evaluates the overall quality of evidence based on five key domains: risk of bias, inconsistency, indirectness, imprecision, and publication bias. Each outcome was initially rated as high-certainty evidence if derived from randomized controlled trials (RCTs), and could be downgraded depending on the presence of serious or very serious limitations in any of the five domains. Conversely, observational studies began as low-certainty evidence and could be upgraded if there was a large effect size, dose–response gradient, or if all plausible confounding would reduce the observed effect.

For this analysis, we assessed outcomes including 5-year and 10-year CR differences, as well as rate ratios. The final certainty ratings were determined through consensus by two independent reviewers using the GRADEpro GDT software (https://www.gradepro.org/, McMaster University and Evidence Prime, Hamilton, ON, Canada), accessed in 2025. High certainty was assigned when findings were consistent across studies, heterogeneity was low (I^2^ < 25%), and no signs of publication bias were detected through funnel plot assessment and Egger’s test.

## 3. Results

### 3.1. Study Selection

**Figure 1:**
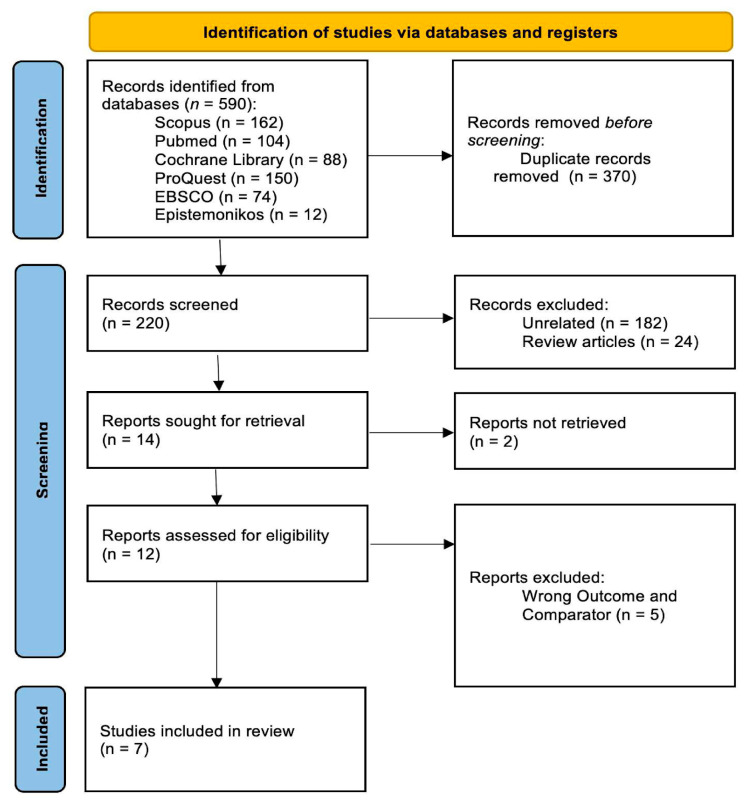
PRISMA Flow Diagram.

Figure 1 presents the PRISMA flow diagram of the study selection process where a total of 590 records were identified from multiple databases, including 162 from Scopus, 104 from PubMed, 88 from the Cochrane Library, 150 from ProQuest, 74 from EBSCO, and 12 from Epistemonikos. After the removal of 370 duplicate records, 220 studies remained for title and abstract screening. Of these, 206 studies were excluded, primarily because they were not relevant to the research question (n = 182) or were review articles (n = 24). This process resulted in 14 studies eligible for full-text assessment; however, only 12 full-text articles were successfully retrieved.

Following detailed evaluation, 5 studies were further excluded due to inappropriate outcome measures or the use of non-relevant comparators [19,20,21,22,23]. Ultimately, 7 studies met all predefined inclusion criteria and were included in the final systematic review. The study selection process is summarized in the PRISMA flow diagram (Figure 1). Subsequently, the included studies underwent risk-of-bias assessment using appropriate methodological tools, with the findings presented in the following sections.

### 3.2. Study Characteristics

This systematic review includes seven studies with a total of 11,391 participants, covering research conducted in the Netherlands, the United States, Australia, and Denmark. These studies used a mix of retrospective and prospective cohort designs, as well as case–control approaches, to explore the impact of germline mutations (BRCA1/2, CHEK2, and ATM) and RT on CBC risk. Most studies focused on BRCA1/2 carriers, while others examined CHEK2*1100delC and ATM rare missense variants. A key research question was whether receiving RT after primary BC treatment influenced the likelihood of developing CBC in these high-risk populations. Across studies, 3D-conformal radiotherapy (3D-CRT) was the primary technique used, though the reported RT doses varied, with some studies specifying dose ranges and others leaving them unspecified.

Participants in these studies had a mean age at first BC diagnosis ranging from 40 to 43 years. Only some studies reported ER and HER2 status. With a range of study designs and international representation, this review offers a well-rounded perspective on how genetic mutations and treatment choices influence long-term BC outcomes. By bringing together findings from different healthcare settings, it provides a clearer picture of how RT might impact CBC risk in mutation carriers. Details on study characteristics, including population, genetic profiles, RT exposure, and key outcomes are available in Table 1.

### 3.3. Risk of Bias Assessment

**Figure 2:**
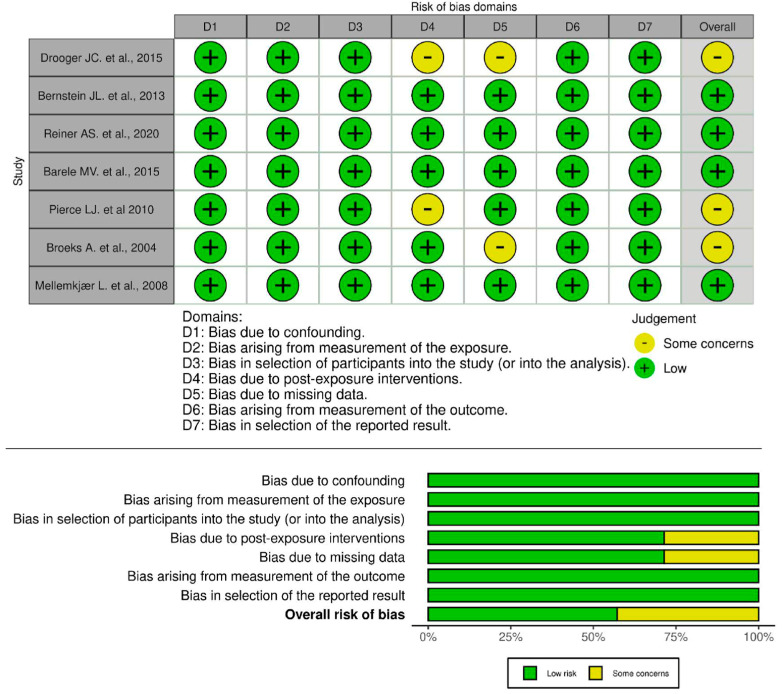
Risk of Bias Assessment [23,24,25,26,27,28,29].

The risk of bias assessment indicates that most studies exhibit a low risk of bias across multiple domains. The overall risk of bias reflects this pattern, with approximately 75% of studies classified as low risk and around 25% exhibiting some concerns. The bar chart visualization further supports this finding, showing that while most domains maintain a low risk of bias, D4 and D5 contribute to potential methodological concerns. In summary, the majority of studies demonstrate strong methodological quality with minimal risk of bias, though minor concerns related to post-exposure interventions and missing data suggest areas for improvement to enhance the reliability of the findings (Figure 2).

### 3.4. Five Years Cumulative Risk of CBC

**Figure 3:**
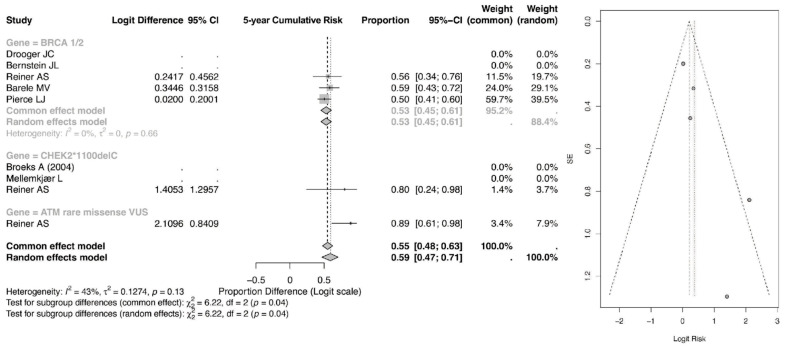
Forest and Funnel Plot for 5-year CR of CBC [23,24,25,26,27,28,29].

The pooled 5-year event proportion derived from the logit-transformed model for BRCA1/2 mutation carriers treated with radiotherapy was 0.55 (95% CI: 0.48–0.63) under the common-effect model and 0.55 (95% CI: 0.45–0.61) under the random-effects model as shown in Figure 3. Importantly, these values reflect model-based back-transformed proportions rather than direct estimates of absolute cumulative incidence. As such, they should be interpreted as relative event proportions within the pooled dataset, not as true population-level 5-year cumulative risks, which are typically substantially lower in clinical practice.

In comparison, the ATM rare missense variant subgroup demonstrated a higher pooled event proportion of 0.89 (95% CI: 0.61–0.98) in the common-effect model and 0.89 (95% CI: 0.24–0.98) in the random-effects model, while the CHEK2 subgroup showed a pooled estimate of 0.80 (95% CI: 0.24–0.98) and 0.80 (95% CI: 0.34–0.76), respectively. Although these findings suggest a comparatively higher relative event burden among ATM and CHEK2 carriers within the analyzed cohorts, caution is warranted in interpreting these values as absolute 5-year risks.

Overall, the pooled estimate across all genetic subgroups was 0.55 (95% CI: 0.47–0.71). The observed heterogeneity (I^2^ = 43%) indicates moderate between-study variability, and subgroup differences were statistically significant (*p* = 0.04), suggesting that mutation type may influence relative event occurrence. However, further validation using directly reported cumulative incidence data is needed before drawing firm clinical conclusions regarding absolute risk.

### 3.5. Ten-Year Cumulative Risk of CBC

**Figure 4:**
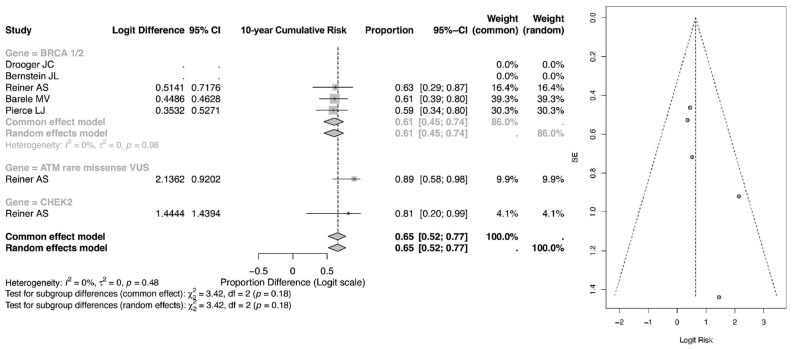
Forest and Funnel Plot for 10-year CR of CBC [23,24,25,26,27].

The pooled 10-year event proportion, shown in Figure 4, derived from the logit-transformed model for BRCA1/2 mutation carriers treated with radiotherapy was 0.65 (95% CI: 0.52–0.77) under both the common-effect and random-effects models. Importantly, these estimates reflect back-transformed pooled proportions within the included datasets rather than direct measures of absolute 10-year cumulative incidence. As such, they should be interpreted cautiously and not equated with population-level 10-year risk, which is typically reported at substantially lower percentages in longitudinal cohort studies.

Within genetic subgroups, carriers of ATM rare missense variants demonstrated a pooled proportion of 0.81 (95% CI: 0.20–0.99), while the CHEK2 subgroup showed an estimate of 0.63 (95% CI: 0.29–0.87). Although these values suggest a relatively greater event burden among ATM carriers within the analyzed cohorts, the wide confidence intervals—particularly in ATM and CHEK2 groups—reflect limited sample size and reduced precision.

The overall pooled 10-year estimate across all subgroups was 0.65 (95% CI: 0.52–0.77), with no observed heterogeneity (I^2^ = 0%). Subgroup differences were not statistically significant (*p* = 0.18), indicating that between-gene variation in pooled proportions at 10 years should be interpreted cautiously. While the 10-year pooled proportions appear numerically higher than the 5-year estimates, this likely reflects the expected temporal accumulation of events rather than a true doubling of absolute clinical risk. Further confirmation using directly reported cumulative incidence data would be required to establish clinically meaningful long-term risk differences.

### 3.6. Rate Ratio of CBC

**Figure 5:**
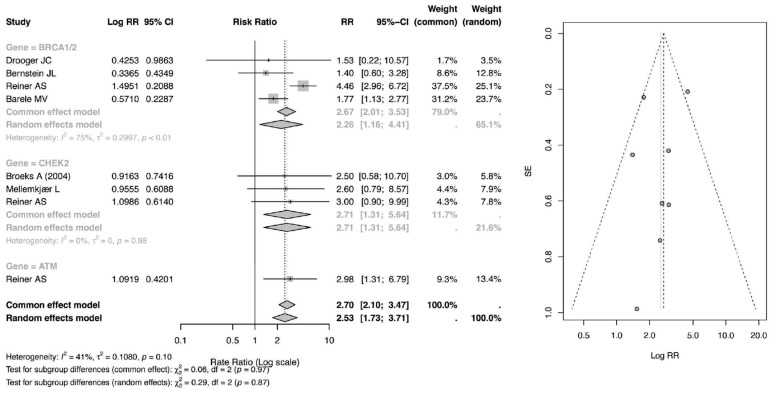
Forest and Funnel Plot for RR of CBC [23,24,25,26,28,29].

The risk ratio (RR) meta-analysis, as in Figure 5, evaluated the relative risk of secondary malignancy among patients receiving radiotherapy (RT) compared with those not exposed to RT across genetic subgroups. Among BRCA1/2 mutation carriers, the pooled RR was 2.67 (95% CI: 2.01–3.53) under the common-effect model and 2.26 (95% CI: 1.16–4.41) under the random-effects model, indicating a statistically significant increase in relative risk following RT. Moderate heterogeneity was observed within this subgroup (I^2^ = 75%), suggesting variability across contributing studies.

For CHEK2 carriers, the pooled RR was 2.71 (95% CI: 1.31–5.64), demonstrating a significant elevation in relative risk, although estimates were derived from a smaller number of studies with relatively wide confidence intervals. Similarly, patients with ATM rare missense variants exhibited an RR of 2.98 (95% CI: 1.31–6.79), suggesting nearly a threefold increase in relative risk; however, this estimate was based on a single study and should therefore be interpreted with caution.

Overall, the pooled RR across all genetic subgroups was 2.70 (95% CI: 2.10–3.47) in the common-effect model and 2.53 (95% CI: 1.73–3.71) in the random-effects model, confirming a statistically significant association between RT exposure and increased relative risk of secondary malignancy. Between-subgroup differences were not statistically significant (*p* = 0.97 for common-effect; *p* = 0.87 for random-effects), indicating that while point estimates vary numerically across genes, the magnitude of RT-associated risk does not differ significantly by mutation type within the limits of available data.

### 3.7. Summary of Findings

This meta-analysis demonstrated significant associations in both cumulative risk (CR) and relative risk (RR) across germline mutation subgroups, as summarized in Table 2. The 5-year CR was significantly elevated among BRCA1/2 carriers (0.55, 95% CI: 0.48–0.63, *p* = 0.04) as well as in the overall population (0.55, 95% CI: 0.47–0.71, *p* = 0.01), indicating an increased early risk of contralateral breast cancer (CBC). This elevated risk appeared to persist over time, as reflected by the significant 10-year CR in the overall analysis (0.65, 95% CI: 0.52–0.77, *p* = 0.02). In addition, ATM mutation carriers demonstrated a markedly increased risk, with an RR of 2.98 (95% CI: 1.31–6.79), suggesting nearly a threefold increase in secondary malignancy. Consistently, pooled estimates across all genetic subgroups confirmed a significantly elevated risk, with an RR of 2.70 (95% CI: 2.10–3.47, *p* = 0.001) in the common-effects model and 2.53 (95% CI: 1.73–3.71, *p* = 0.001) in the random-effects model.

Despite these significant findings, the interpretation of the pooled estimates should be approached with caution due to notable heterogeneity across the included studies. Although all studies primarily employed 3D-conformal radiotherapy (3D-CRT), detailed reporting of radiation exposure—particularly to the contralateral breast—was limited and inconsistent. Only a minority of studies provided quantitative dose estimates, which restricts the ability to establish a clear dose–response relationship and may contribute to variability in observed risk estimates.

Furthermore, substantial heterogeneity arises from differences in follow-up duration, which is particularly relevant given the long latency period associated with radiation-induced malignancies. Studies with shorter follow-up may underestimate the true incidence of CBC, whereas longer-term studies are more likely to capture late events. In addition, variability in genetic composition across studies further complicates interpretation. BRCA1/2 mutation carriers constituted the majority of the population, while CHEK2 and ATM mutation carriers were relatively underrepresented, often with small sample sizes, thereby limiting the robustness of subgroup-specific conclusions.

Finally, differences in patient and tumor characteristics—including age at diagnosis, hormonal receptor status, and HER2 expression—were not consistently reported across studies. These factors may independently influence CBC risk and interact with radiation exposure, contributing additional layers of heterogeneity. Taken together, these variations in radiotherapy parameters, follow-up duration, genetic profiles, and clinical characteristics may affect both the magnitude and direction of the pooled estimates. Therefore, while our findings suggest an increased risk of CBC associated with radiotherapy in mutation carriers, they should be interpreted within the context of these limitations, underscoring the need for more standardized, mutation-specific, and dosimetry-informed future studies.

The five- and ten-year cumulative risk (CR) findings were rated as moderate certainty, reflecting consistent pooled estimates and statistically significant overall effects. Although heterogeneity was low to moderate (I^2^ ranging from 0% to 43%) and no strong publication bias was detected, the certainty was limited by the observational design of the included studies and imprecision within certain genetic subgroups, particularly ATM and CHEK2, where small sample sizes and wide confidence intervals were observed. The rate ratio (RR) findings were also classified as moderate certainty, supported by a strong and statistically significant pooled effect size across analyses. However, moderate-to-high heterogeneity in some subgroups and limited subgroup-level precision prevented a high-certainty classification. These ratings suggest that the 10-year CR and RR results demonstrate reasonably robust and clinically meaningful associations, whereas the 5-year CR findings should be interpreted with some caution due to residual variability and subgroup imprecision (Table 3).

## 4. Discussion

Our meta-analysis demonstrates a significant association between RT and an increased risk of CBC in primary BC patients carrying BRCA1/2, CHEK2, and ATM mutations. These findings highlight the importance of understanding the interaction between genetic predisposition and treatment modalities in BC with germline mutation management [30].

### 4.1. Risk of CBC Development in Germline Mutation Carriers

Individuals with protein-truncating variants (PTVs) and pathogenic or likely pathogenic missense variants (MSVs) in BRCA1 and BRCA2 have been previously reported to face an approximately 2-fold and 3-fold risk, respectively, of developing CBC. A study by Borg et al. (2010) found that patients with mutations in the 3′ region of BRCA1 had a lower frequency of CBC compared to those with mutations in the 5′ or middle regions of the gene [11]. Premature termination codons (PTCs) are usually degraded by nonsense-mediated mRNA decay (NMD), preventing the production of truncated or non-functional proteins. However, if a premature stop codon is located less than 50 nucleotides from the last exon–exon junction (EEJ) or occurs within the final exon, it may evade NMD and still be translated into a partially functional protein. Mutations occurring at BRCA1 codon 1807 or later may produce proteins with some residual function. Therefore, mutations in the 3′ region of BRCA1 may have a less severe impact compared to mutations in the 5′ region, as they can escape the NMD mechanism, allowing the production of proteins that still retain some function. This could explain why fewer CBC patients have mutations in the 3′ region of BRCA1 compared to other regions [31].

Yadav et al. (2023) revealed that including additional truncating variants in CHEK2, beyond the well-known c.1100delC mutation, strengthened the association with an increased risk of CBC [4]. This suggests that all truncating mutations in CHEK2 likely contribute to CBC risk, rather than just the c.1100delC variant alone. However, when missense PVs were also included in the analysis, the previously observed strong correlation between CHEK2 mutations and CBC risk became weaker. This raises uncertainty regarding whether missense PVs in CHEK2 genuinely contribute to CBC risk, or if the increased risk is predominantly driven by truncating mutations [32].

Our study found that RT in ATM mutation did not significantly elevate the risk of CBC occurrence. This aligns with a study by Yadav et al. (2023) which concluded that carriers of germline ATM PVs did not show a significantly increased risk of CBC overall (HR: 1.2; 95% CI: 0.6–2.6; *p* = 0.56) [4]. Interpreting ATM mutations is more challenging than other BC susceptibility genes because the cancer risk varies depending on the specific mutation. Many high-risk ATM mutations are missense mutations, which can be difficult to distinguish from normal genetic variations (benign polymorphisms). Additionally, ATM mutations can be either truncating or non-truncating, and research suggests that non-truncating mutations may be associated with a higher BC risk [33].

In a previous study by Morraz et al. (2023), TP53 PTVs and pathogenic/likely pathogenic MSVs combined were associated with an eightfold increase in CBC risk [30]. While PALB2 PV carriers did not show a significantly higher overall CBC risk, those with ER-negative primary BC had a notably increased risk (HR: 2.9; 95% CI: 1.4–6.4; *p* = 0.006) compared to ER-positive BC where only one case of CBC occurred out of 54 carriers [4].

### 4.2. Risk of CBC in Germline Mutation Carriers with RT

As previously mentioned, this study found a statistically significant risk of developing CBC in mutated CHEK2 and sBRCA1/2 carriers which is different compared to our included studies. Most of them reported that the incidence of CBC is higher in mutated BRCA1/2 who received RT but it is not statistically significant [23,24,26,27]. Bernstein et al. (2013) found a 40% higher risk of CBC in deleterious BRCA1/2 patients with RT, though not significant [24]. Radiotherapy with a radiation dose < 1 Gy had an increased risk of CBC in mutated patients (RR = 1.9). However, higher radiation dose was not found to be statistically significant with greater CBC risk [24]. Reiner et al. (2020) additionally reported that CBC development is not statistically significant in CHEK2*1100delC and pathogenic or likely pathogenic (PLP) ATM but interestingly, there is a significant risk in ATM missense carriers who received RT [25]. The incidence of CBC after RT in patients with CHEK2*1100delC mutation carriers is higher than those who did not receive RT (OR = 2.5), although it is not significant [19].

For high-risk patients, CBC risk can be reduced by at least 90% through preventive measures like Contralateral Prophylactic Mastectomy (CPM) [10]. American Society of Clinical Oncology Annual Meeting in 2013 showed a statistically significant survival benefit for patients who underwent prophylactic mastectomy, with a 10-year overall survival of 90%, compared to 80% in those who did not undergo mastectomy in BC patients [34]. This contradicts with a study by Valachis et al. (2014) who found no significant survival difference between patients who underwent contralateral prophylactic mastectomy and those who did not [10]. However, the study was limited by a small sample size and a relatively short median follow-up compared to the previous findings. Additionally, the analysis showed that adjuvant chemotherapy did not significantly impact CBC risk (RR 0.90, 95% CI 0.66–1.22). On the other hand, adjuvant tamoxifen use and oophorectomy were associated with a nearly 50% reduction in CBC risk [10].

### 4.3. Current Guidelines for BC with Germline Mutation

Radiotherapy (RT) remains a cornerstone of breast cancer management and is recommended based on multiple clinicopathological factors, including tumor stage, nodal involvement, surgical margins, and type of surgery performed. Following breast-conserving surgery (BCS), whole-breast irradiation is widely accepted as a standard approach to reduce the risk of ipsilateral local recurrence, while accelerated partial-breast irradiation may be considered in carefully selected patients. In the postmastectomy setting, RT is generally indicated for individuals at higher risk of locoregional recurrence, particularly those with significant nodal burden or other adverse pathological features. Importantly, the presence of germline BRCA1/2 mutations should not be interpreted as an absolute contraindication to RT. Rather, treatment decisions should be individualized, taking into account patient preferences, oncologic risk, and the potential long-term implications, including the risk of subsequent primary malignancies. Current evidence suggests that appropriately selected patients with BRCA1/2 mutations may still be candidates for breast-conserving approaches with adjuvant RT, provided that thorough counseling is undertaken [35].

For patients with germline BRCA1/2-mutated breast cancer, systemic therapy plays a critical role and should be guided by disease stage and risk stratification. In the metastatic setting, poly (ADP-ribose) polymerase (PARP) inhibitors, such as olaparib and talazoparib, have demonstrated superior progression-free survival compared with standard single-agent chemotherapy in patients with HER2-negative disease. In addition, the OlympiA trial provided pivotal evidence supporting the use of adjuvant olaparib in patients with high-risk, HER2-negative early breast cancer harboring germline BRCA1/2 pathogenic variants. In this study, one year of adjuvant olaparib following completion of local treatment and (neo)adjuvant chemotherapy significantly improved invasive disease-free survival and distant disease-free survival, with subsequent analyses also demonstrating a meaningful overall survival benefit [35,36,37,38]. Collectively, these findings highlight the evolving role of targeted systemic therapies and underscore the importance of integrating genomic information into treatment decision-making for this patient population.

### 4.4. Optimizing Radiotherapy Dose

Radiotherapy (RT) remains a cornerstone in the management of primary breast cancer; however, concerns persist regarding its potential contribution to contralateral breast cancer (CBC), particularly in patients with germline mutations such as BRCA1/2, CHEK2, and ATM. One key consideration in addressing this risk is accurate characterization and minimization of radiation exposure to the contralateral breast. In the study by Bernstein et al., the reported mean radiation dose of 1.1 Gy (range 0.02–6.2 Gy) represents an estimated dose to the contralateral breast, rather than the direct therapeutic dose delivered to the primary tumor [24]. The analysis demonstrated a modestly increased risk of CBC among individuals with estimated exposures exceeding 1 Gy (RR = 1.2, 95% CI = 1.0–1.6), suggesting a potential dose–response relationship. These findings underscore the importance of minimizing unintended radiation exposure, particularly in patients with underlying genetic susceptibility to radiation-induced malignancies.

Advances in modern RT techniques have focused on improving dose conformity while reducing scatter radiation to adjacent normal tissues, including the contralateral breast. Techniques such as IMRT, VMAT, and proton therapy enable more precise dose and may contribute to lowering off-target exposure. Consequently, individualized RT planning—taking into account genetic risk factors, tumor characteristics, and anatomical considerations—is essential to balance optimal oncologic outcomes with long-term safety.

### 4.5. Advances in Radiotherapy Techniques for Breast Cancer

Radiotherapy (RT) remains a fundamental component of breast cancer management, with well-established evidence demonstrating its role in improving local control and, in selected patient groups, contributing to survival benefits. Following breast-conserving surgery, RT significantly reduces the risk of ipsilateral recurrence and is considered a standard of care. Similarly, in the postmastectomy setting, RT provides meaningful reductions in locoregional recurrence, particularly in patients with high-risk pathological features. These therapeutic benefits underscore the critical importance of RT in achieving optimal oncologic outcomes.

At the same time, several RT techniques have been developed to optimize target coverage while minimizing unintended exposure to surrounding normal tissues. Three-dimensional conformal radiotherapy (3D-CRT) remains a widely used standard approach, utilizing multiple shaped beams to conform to the target volume and limit dose to adjacent organs at risk [39,40]. However, the dose delivered to the contralateral breast is influenced not only by the technique employed but also by tumor location, patient anatomy, and beam configuration. Even with conformal approaches, low-dose radiation may still reach non-target regions, including the ipsilateral breast outside the treatment field and the contralateral breast, which may contribute to long-term risks.

Additionally, strategies that reduce the irradiated volume, such as accelerated partial breast irradiation (APBI), may further limit exposure to both the contralateral breast and non-target regions of the ipsilateral breast [41]. Brachytherapy, as a form of APBI, enables highly localized dose delivery with rapid dose fall-off, although its use remains limited to carefully selected patients [42].

More advanced photon-based techniques, such as intensity-modulated radiotherapy (IMRT) and volumetric modulated arc therapy (VMAT), offer improved dose homogeneity and target conformity. However, these approaches may increase the overall integral dose delivered to non-target tissues compared with 3D-CRT, and therefore do not necessarily reduce radiation exposure to the contralateral breast [40]. This consideration is particularly relevant in patients with germline mutations such as BRCA1/2, CHEK2, or ATM, where minimizing radiation to uninvolved breast tissue may be a priority. In contrast, proton beam therapy has emerged as an alternative modality with distinct dosimetric advantages, characterized by the Bragg peak effect, which enables maximal dose deposition within the target while substantially reducing exit dose to surrounding tissues. This property may allow for lower radiation exposure to critical structures such as the heart, lungs, and contralateral breast, particularly in selected cases such as left-sided breast cancer [43].

Taken together, these considerations highlight the importance of balancing the proven oncologic benefits of RT against its potential long-term risks, particularly in genetically predisposed populations. While RT remains a highly effective modality for improving disease control, individualized treatment planning is essential. Clinicians must carefully weigh the magnitude of benefit in local control and survival against the potential risk of radiation-induced secondary malignancies, including contralateral breast cancer. As such, shared decision-making—incorporating genetic risk, tumor characteristics, and patient preferences—plays a central role in optimizing treatment strategies and ensuring that both short-term efficacy and long-term safety are appropriately addressed.

### 4.6. Study Strength and Limitation

This meta-analysis has several important limitations that should be carefully considered when interpreting the findings. First, all included studies were observational in design (cohort and case–control), which introduces the potential for selection bias and residual confounding, despite statistical adjustments. Second, there was substantial heterogeneity across studies in terms of design, sample size, radiotherapy (RT) techniques, and duration of follow-up. In particular, variations in radiation delivery and the limited availability of detailed dosimetric data—especially regarding exposure to the contralateral breast—restrict the ability to establish clear dose–response relationships. More consistent and standardized reporting of radiation parameters is therefore essential to better understand the true magnitude of RT-associated risk.

A key limitation of the current evidence is the relatively small number of studies evaluating specific germline mutation subgroups. While BRCA1/2 carriers were more frequently represented, data on CHEK2 and ATM mutation carriers were limited and often derived from small sample sizes, reducing the precision and generalizability of subgroup-specific risk estimates. In addition, genetic data were inconsistently reported, with limited differentiation between mutation types (e.g., BRCA1 versus BRCA2, or truncating versus missense variants), further constraining mutation-specific interpretation. The clinical relevance of metachronous contralateral breast cancer is also difficult to fully contextualize in the absence of key information such as latency period following radiation exposure, tumor characteristics, and the impact of systemic therapies, including chemotherapy and endocrine treatment.

Furthermore, although our findings suggest an increased risk of contralateral breast cancer associated with RT in genetically predisposed populations, causal inference remains challenging. Unmeasured or incompletely reported confounders—including hormonal factors, lifestyle variables, and treatment-related differences—may have influenced the observed associations.

Future research should prioritize well-designed prospective studies with larger, mutation-specific cohorts and longer follow-up to better capture the latency of radiation-induced malignancies. Standardization of RT reporting, particularly with respect to detailed dosimetry to the contralateral breast, is crucial. In addition, integrating genomic stratification with comprehensive clinical and treatment data will be essential to refine risk estimates and support more precise, individualized treatment strategies. Such efforts will ultimately enhance the clinical applicability of evidence and improve decision-making for patients with hereditary breast cancer.

## 5. Conclusions

This systematic review and meta-analysis suggest that radiotherapy (RT) is associated with an increased risk of contralateral breast cancer (CBC) among patients with germline mutations, particularly in carriers of BRCA1/2 and CHEK2 variants. However, as all included studies were observational in design, these findings reflect statistical associations rather than definitive causal effects. Residual confounding—including differences in age at diagnosis, systemic therapy exposure, endocrine treatment, and surveillance intensity—may have influenced the observed risk estimates.

Given these considerations, individualized treatment decision-making remains essential for patients with hereditary breast cancer predisposition. The potential long-term risk of CBC should be carefully weighed against the established survival benefits of RT in primary breast cancer management. Future prospective studies incorporating detailed dosimetric data, robust adjustment for confounders, and dose–response analyses are needed to clarify the magnitude and biological basis of RT-associated CBC risk. In the interim, optimization of RT techniques, including modern conformal planning and dose minimization strategies, may help mitigate potential long-term risks in genetically susceptible populations.

## Figures and Tables

**Table 1:** Study Characteristics.

No	Studies	Country	Design	Total	Germline Assessed	Carrier (n)		Mean Age at First PBC		RT Technique	Radiation Dose to Contralateral (Gy)		ER Status		HER2 Status	
													CBC		CBC	
						RT	No-RT	Mean	SD		Mean	Min–Max	+	−	+	−
BRCA1/2																
1	Drooger JC [23]	Netherlands	Retrospective Cohort	691	BRCA1/2	434	252	NR	NR	3D-CRT	NR	NR	227	348	17	236
2	Bernstein JL [24]	USA	Case–Control	1802	BRCA1/2	109	49	NR	NR	3D-CRT	1.1	0.02–6.20	NR	NR	NR	NR
3	Reiner AS [25]	USA	Case–Control	2107	BRCA1/2, CHEK2, ATM	127	58	NR	NR	3D-CRT	NR	NR	NR	NR	NR	NR
4	Barele MV [26]	Netherlands	Prospective Cohort	3602	BRCA1/2	2297	1305	41.93	9.88	3D-CRT	NR	NR	1060	1274	124	1365
5	Pierce LJ [27]	Australia	Retrospective Cohort	665	BRCA1/2	103	250	43	9.8	3D-CRT	NR	NR	216	449	NR	NR
CHEK2*1100delC																
6	Broeks A [28]	Netherlands	Retrospective Cohort	424	CHEK2	13	2	NR	NR	3D-CRT	NR	NR	NR	NR	NR	NR
7	Mellemkjær L [29]	USA, Denmark	Case–Control	2103	CHEK2	13	4	40.8	NR	3D-CRT	1.29	0.03–4.68	338	193	NR	NR
3	Reiner AS [25]	USA	Case–Control	2107	BRCA1/2, CHEK2, ATM	4	13	NR	NR	3D-CRT	NR	NR	NR	NR	NR	NR
ATM rare missense VUS																
3	Reiner AS [25]	USA	Case–Control	2107	BRCA1/2, CHEK2, ATM	4	13	NR	NR	3D-CRT	NR	NR	NR	NR	NR	NR

NR: Not Reported; RT: Radiotherapy; No-RT: No Radiotherapy; PBC: Primary Breast Cancer.

**Table 2:** Summary of Findings.

	BRCA1/2		ATM		CHEK2		Overall		
	CR/RR 95%CI [Lower–Upper]	*p*-Value	CR/RR 95%CI [Lower–Upper]	*p*-Value	CR/RR 95%CI [Lower–Upper]	*p*-Value	CR/RR 95%CI [Lower–Upper]	*p*-Value	*I* ^2^
*Common Effects Model*									
5-Year Cumulative Risk Differences	0.55 [0.48–0.63] (n = 3)	0.04 *	0.89 [0.61–0.98] (n = 1)	NA	0.80 [0.24–0.98] (n = 1)	NA	0.55 [0.47–0.71] (n = 5)	0.01 *	43%
10-Year Cumulative Risk Differences	0.65 [0.52–0.77] (n = 3)	0.05	0.81 [0.20–0.99] (n = 1)	NA	0.63 [0.29–0.87] (n = 1)	NA	0.65 [0.52–0.77] (n = 5)	0.02 *	0%
Rate Ratio	1.53 [0.22–10.57] (n = 4)	0.03 *	2.98 [1.31–6.79] (n = 1)	NA	3.00 [0.90–9.99] (n = 3)	0.02 *	2.70 [2.10–3.47] (n = 8)	0.001 *	41%
*Random Effects Model*									
5-Year Cumulative Risk Differences	0.55 [0.45–0.61] (n = 3)	0.04 *	0.89 [0.24–0.98] (n = 1)	NA	0.80 [0.34–0.76] (n = 1)	NA	0.55 [0.47–0.71] (n = 5)	0.01 *	43%
10-Year Cumulative Risk Differences	0.65 [0.45–0.74] (n = 3)	0.05	0.81 [0.20–0.99] (n = 1)	NA	0.63 [0.29–0.87] (n = 1)	NA	0.65 [0.52–0.77] (n = 5)	0.02 *	0%
Rate Ratio	1.53 [0.22–10.57] (n = 4)	0.03 *	2.98 [1.31–6.79] (n = 1)	NA	3.00 [0.90–9.99] (n = 3)	0.02 *	2.53 [1.73–3.71] (n = 8)	0.001 *	41%

* significant (*p* < 0.05); CR: Cumulative Risk; RR: Rate Ratio; CI: Confidence Intervals; NA: Not Available.

**Table 3:** Summary of Grade Analysis.

Outcome	Final Certainty Rating	Key Justification
5-Year Cumulative Risk Differences (CR)	Moderate	Evidence derived from observational studies (initially low certainty). Upgraded due to consistent direction of effect across models and statistically significant pooled estimates. However, moderate heterogeneity (I^2^ = 43%) and limited subgroup precision (ATM and CHEK2 based on single studies with wide CIs) prevent a high-certainty rating.
10-Year Cumulative Risk Differences (CR)	Moderate	Observational design limits baseline certainty. Although heterogeneity was absent (I^2^ = 0%) and overall results were statistically significant and consistent, subgroup analyses were based on small sample sizes with wide confidence intervals, introducing imprecision.
Rate Ratio (RR) of CBC	Moderate	Large and statistically significant pooled effect size (RR ~2.5–3.0) supports upgrading from low to moderate certainty. However, moderate-to-high heterogeneity (I^2^ up to 75% in BRCA1/2 subgroup), small subgroup sample sizes, and wide CIs (particularly ATM and CHEK2) prevent classification as high certainty.

## Data Availability

All data supporting the findings presented in this study are included as part of the article, and there is no need for additional source data.

## Supplementary Materials

The following supporting information can be downloaded at https://www.mdpi.com/article/10.3390/cancers18071106/s1, Supplementary File S1: Data Extraction; Supplementary File S2: Risk of Bias Asessment.

## Abbreviations

The following abbreviations are used in this manuscript:

ATM	Ataxia telangiectasia mutated
BC	Breast cancer
BRCA1	Breast cancer gene 1
BRCA2	Breast cancer gene 2
CBC	Contralateral breast cancer
CHEK 2	Checkpoint kinase 2
ER	Estrogen receptor
HER2	Human epidermal growth factor receptor 2
PBC	Primary breast cancer
RT	Radiotherapy
